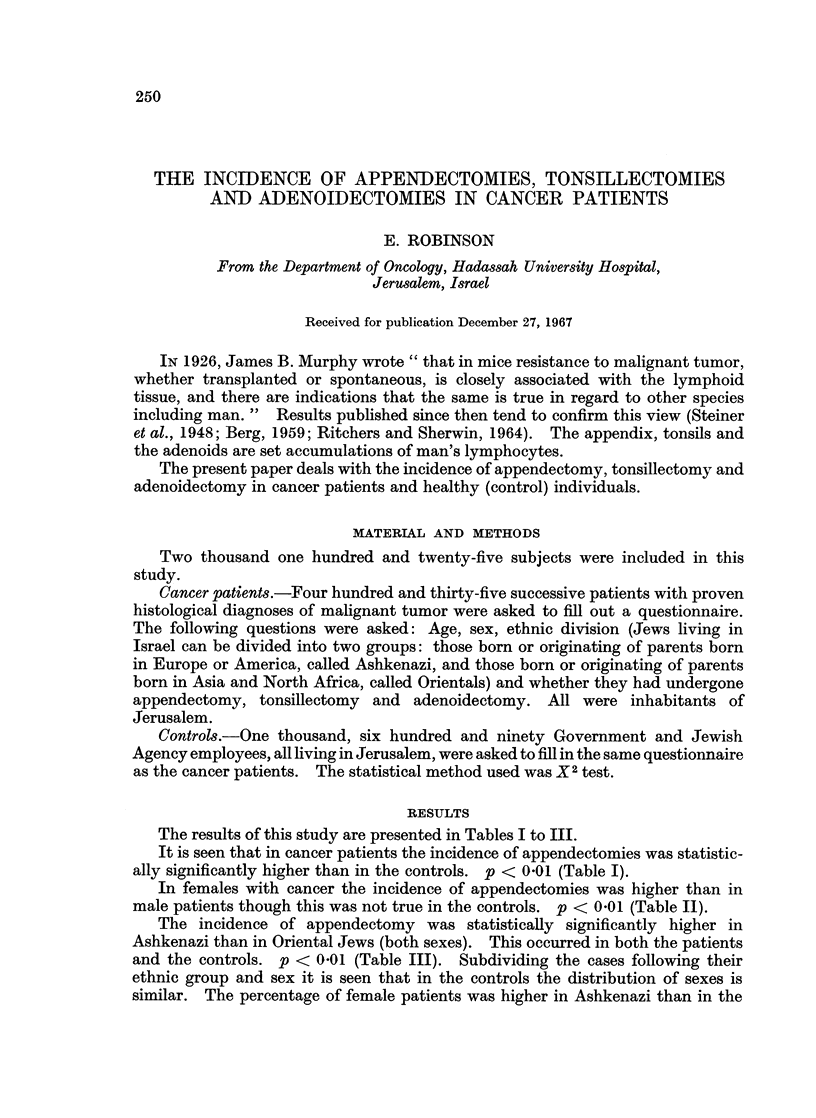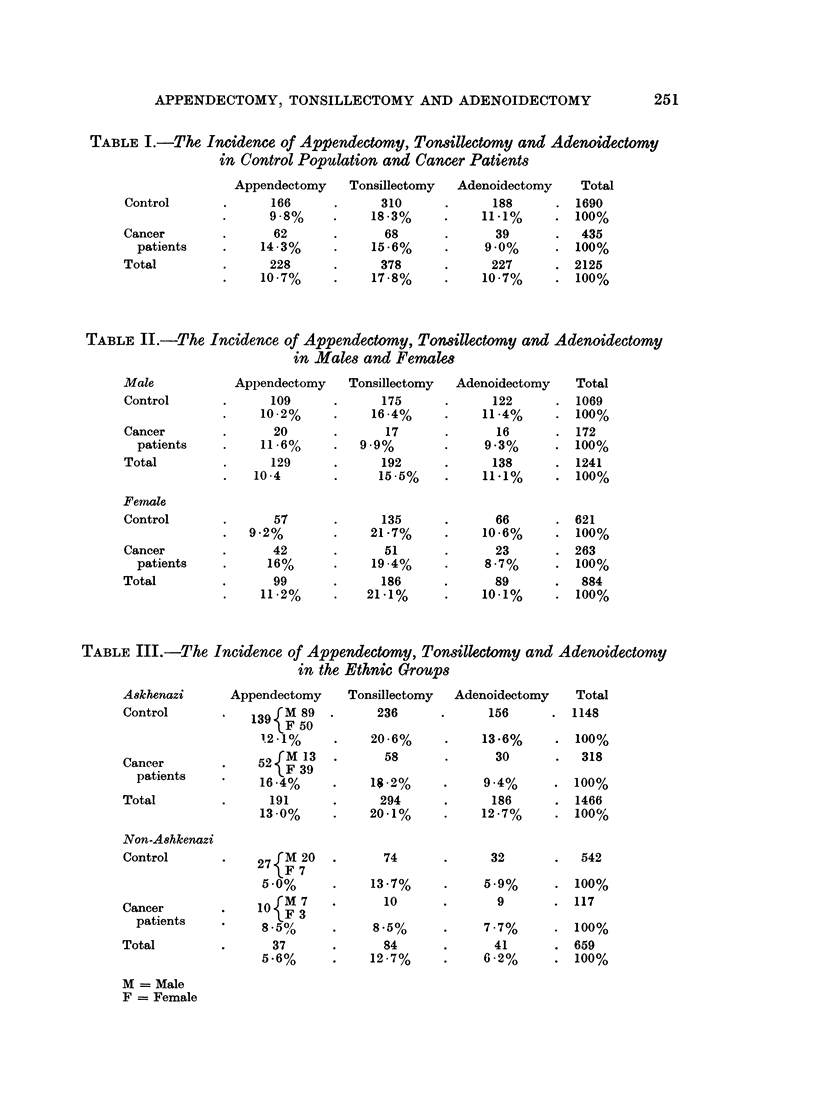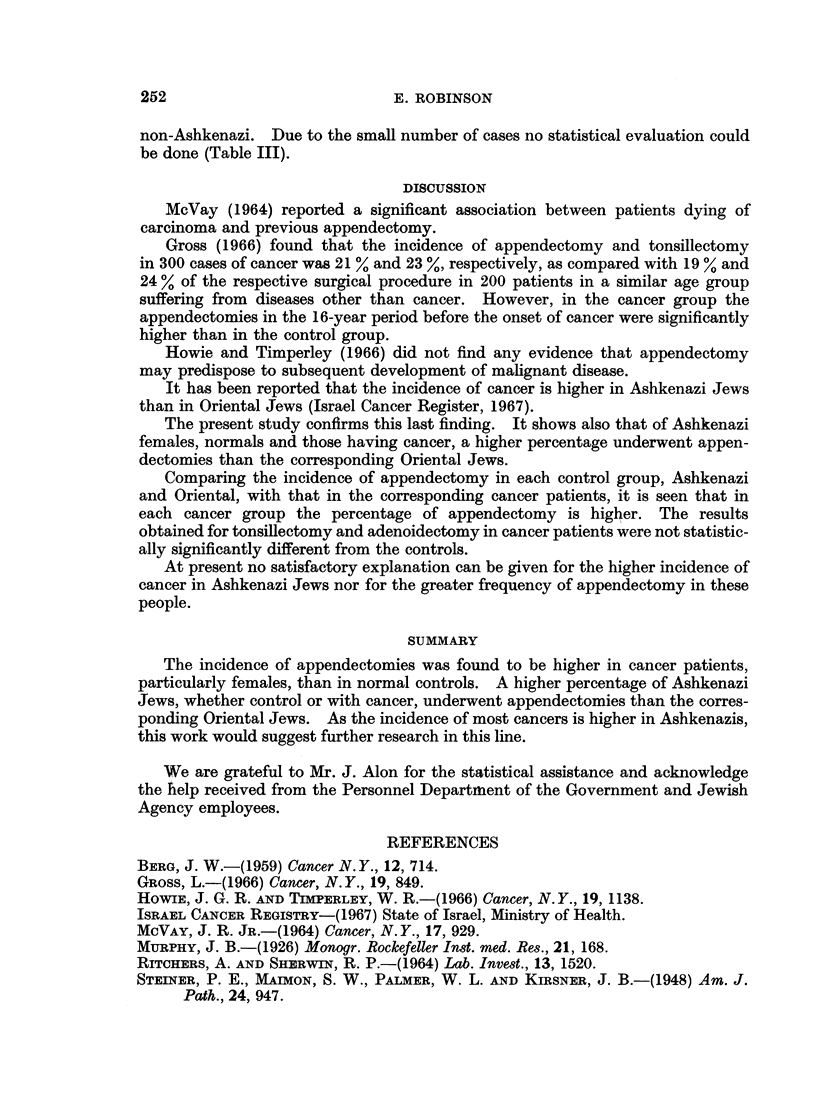# The incidence of appendectomies, tonsillectomies and adenoidectomies in cancer patients.

**DOI:** 10.1038/bjc.1968.32

**Published:** 1968-06

**Authors:** E. Robinson


### THE INCIDENCE OF APPENDECTOMIES, TONSILLECTOMIES AND ADENOIDECTOMIES IN CANCER PATIENTS

E. ROBINSON

*From the Department of Oncology, Hadassah University Hospital, Jerusalem, Israel*

Received for publication December 27, 1967

In 1926, James B. Murphy wrote " that in mice resistance to malignant tumor, whether transplanted or spontaneous, is closely associated with the lymphoid tissue, and there are indications that the same is true in regard to other species including man. " Results published since then tend to confirm this view (Steiner *et al.*, 1948; Berg, 1959; Ritchers and Sherwin, 1964). The appendix, tonsils and the adenoids are set accumulations of man's lymphocytes.

The present paper deals with the incidence of appendectomy, tonsillectomy and adenoidectomy in cancer patients and healthy (control) individuals.

### MATERIAL AND METHODS

Two thousand one hundred and twenty-five subjects were included in this study.

*Cancer patients.*—Four hundred and thirty-five successive patients with proven histological diagnoses of malignant tumor were asked to fill out a questionnaire. The following questions were asked: Age, sex, ethnic division (Jews living in Israel can be divided into two groups: those born or originating of parents born in Europe or America, called Ashkenazi, and those born or originating of parents born in Asia and North Africa, called Orientals) and whether they had undergone appendectomy, tonsillectomy and adenoidectomy. All were inhabitants of Jerusalem.

*Controls.*—One thousand, six hundred and ninety Government and Jewish Agency employees, all living in Jerusalem, were asked to fill in the same questionnaire as the cancer patients. The statistical method used was $X^2$ test.

### RESULTS

The results of this study are presented in Tables I to III.

It is seen that in cancer patients the incidence of appendectomies was statistically significantly higher than in the controls. $p < 0{\cdot}01$ (Table I).

In females with cancer the incidence of appendectomies was higher than in male patients though this was not true in the controls. $p < 0{\cdot}01$ (Table II).

The incidence of appendectomy was statistically significantly higher in Ashkenazi than in Oriental Jews (both sexes). This occurred in both the patients and the controls. $p < 0{\cdot}01$ (Table III). Subdividing the cases following their ethnic group and sex it is seen that in the controls the distribution of sexes is similar. The percentage of female patients was higher in Ashkenazi than in the

TABLE I.—*The Incidence of Appendectomy, Tonsillectomy and Adenoidectomy in Control Population and Cancer Patients*

| | Appendectomy | Tonsillectomy | Adenoidectomy | Total |
|---|---|---|---|---|
| Control | 166 | 310 | 188 | 1690 |
| | 9·8% | 18·3% | 11·1% | 100% |
| Cancer | 62 | 68 | 39 | 435 |
| patients | 14·3% | 15·6% | 9·0% | 100% |
| Total | 228 | 378 | 227 | 2125 |
| | 10·7% | 17·8% | 10·7% | 100% |

TABLE II.—*The Incidence of Appendectomy, Tonsillectomy and Adenoidectomy in Males and Females*

| *Male* | Appendectomy | Tonsillectomy | Adenoidectomy | Total |
|---|---|---|---|---|
| Control | 109 | 175 | 122 | 1069 |
| | 10·2% | 16·4% | 11·4% | 100% |
| Cancer | 20 | 17 | 16 | 172 |
| patients | 11·6% | 9·9% | 9·3% | 100% |
| Total | 129 | 192 | 138 | 1241 |
| | 10·4 | 15·5% | 11·1% | 100% |
| *Female* | | | | |
| Control | 57 | 135 | 66 | 621 |
| | 9·2% | 21·7% | 10·6% | 100% |
| Cancer | 42 | 51 | 23 | 263 |
| patients | 16% | 19·4% | 8·7% | 100% |
| Total | 99 | 186 | 89 | 884 |
| | 11·2% | 21·1% | 10·1% | 100% |

TABLE III.—*The Incidence of Appendectomy, Tonsillectomy and Adenoidectomy in the Ethnic Groups*

| *Askhenazi* | Appendectomy | Tonsillectomy | Adenoidectomy | Total |
|---|---|---|---|---|
| Control | 139 {M 89, F 50} | 236 | 156 | 1148 |
| | 12·1% | 20·6% | 13·6% | 100% |
| Cancer patients | 52 {M 13, F 39} | 58 | 30 | 318 |
| | 16·4% | 18·2% | 9·4% | 100% |
| Total | 191 | 294 | 186 | 1466 |
| | 13·0% | 20·1% | 12·7% | 100% |
| *Non-Ashkenazi* | | | | |
| Control | 27 {M 20, F 7} | 74 | 32 | 542 |
| | 5·0% | 13·7% | 5·9% | 100% |
| Cancer patients | 10 {M 7, F 3} | 10 | 9 | 117 |
| | 8·5% | 8·5% | 7·7% | 100% |
| Total | 37 | 84 | 41 | 659 |
| | 5·6% | 12·7% | 6·2% | 100% |

M = Male
F = Female non-Ashkenazi. Due to the small number of cases no statistical evaluation could be done (Table III).

### DISCUSSION

McVay (1964) reported a significant association between patients dying of carcinoma and previous appendectomy.

Gross (1966) found that the incidence of appendectomy and tonsillectomy in 300 cases of cancer was 21 % and 23 %, respectively, as compared with 19 % and 24 % of the respective surgical procedure in 200 patients in a similar age group suffering from diseases other than cancer. However, in the cancer group the appendectomies in the 16-year period before the onset of cancer were significantly higher than in the control group.

Howie and Timperley (1966) did not find any evidence that appendectomy may predispose to subsequent development of malignant disease.

It has been reported that the incidence of cancer is higher in Ashkenazi Jews than in Oriental Jews (Israel Cancer Register, 1967).

The present study confirms this last finding. It shows also that of Ashkenazi females, normals and those having cancer, a higher percentage underwent appendectomies than the corresponding Oriental Jews.

Comparing the incidence of appendectomy in each control group, Ashkenazi and Oriental, with that in the corresponding cancer patients, it is seen that in each cancer group the percentage of appendectomy is higher. The results obtained for tonsillectomy and adenoidectomy in cancer patients were not statistically significantly different from the controls.

At present no satisfactory explanation can be given for the higher incidence of cancer in Ashkenazi Jews nor for the greater frequency of appendectomy in these people.

### SUMMARY

The incidence of appendectomies was found to be higher in cancer patients, particularly females, than in normal controls. A higher percentage of Ashkenazi Jews, whether control or with cancer, underwent appendectomies than the corresponding Oriental Jews. As the incidence of most cancers is higher in Ashkenazis, this work would suggest further research in this line.

We are grateful to Mr. J. Alon for the statistical assistance and acknowledge the help received from the Personnel Department of the Government and Jewish Agency employees.

### REFERENCES

Berg, J. W.—(1959) *Cancer N.Y.*, **12**, 714.
Gross, L.—(1966) *Cancer, N.Y.*, **19**, 849.
Howie, J. G. R. and Timperley, W. R.—(1966) *Cancer, N.Y.*, **19**, 1138.
Israel Cancer Registry—(1967) State of Israel, Ministry of Health.
McVay, J. R. Jr.—(1964) *Cancer, N.Y.*, **17**, 929.
Murphy, J. B.—(1926) *Monogr. Rockefeller Inst. med. Res.*, **21**, 168.
Ritchers, A. and Sherwin, R. P.—(1964) *Lab. Invest.*, **13**, 1520.
Steiner, P. E., Maimon, S. W., Palmer, W. L. and Kirsner, J. B.—(1948) *Am. J. Path.*, **24**, 947.